# Insights and modulation of RNA polymerase–dependent R-loop and dsRNA in Fanconi anemia hematopoietic stem cells

**DOI:** 10.1172/jci.insight.192126

**Published:** 2026-02-26

**Authors:** Michihiro Hashimoto, Xiaomin Feng, Jie Bai, Huimin Zeng, Tian Li, Jue Li, Terumasa Umemoto, Paul R. Andreassen, Gang Huang

**Affiliations:** 1 Department of Cell Systems and Anatomy, UT Health San Antonio, San Antonio, Texas, USA.; 2 Department of Pathology and Laboratory Medicine, UT Health San Antonio, Joe R. and Teresa Lozano Long School of Medicine, San Antonio, Texas, USA.; 3 Mays Cancer Center at UT Health San Antonio, San Antonio, Texas, USA.; 4 Laboratory of Stem Cell Engineering, International Research Center for Medical Sciences, Kumamoto University, Kumamoto, Japan.; 5 Pediatric Department, Peking University People’s Hospital, Beijing, China.; 6 Division of Pathology, and; 7 Division of Experimental Hematology and Cancer Biology, Cincinnati Children’s Hospital Medical Center, Cincinnati, Ohio, USA.; 8 Department of Hematology, The First Affiliated Hospital, Zhejiang University School of Medicine, Hangzhou, Zhejiang, China.; 9 Department of Pediatrics, University of Cincinnati College of Medicine, Cincinnati, Ohio, USA.

**Keywords:** Cell biology, Hematology, Hematopoietic stem cells

## Abstract

Fanconi anemia (FA) is the most common BM failure (BMF) syndrome. FA genes have a role in suppressing DNA-RNA hybrids, termed R-loops, which can be generated via transcription mediated by RNA polymerase (RNAP). How these processes, including a role in fate determination of hematopoietic stem cells (HSCs), are related to BMF is largely unknown. Single FA gene KO in mice does not recapitulate most phenotypes observed in patients with FA. Thus, we generated a mouse model for FA by introducing heterozygous *Setd2*, which restricts RNAP-dependent transcription. We showed that FA patient–derived cells and *Setd2^+/–^ Fanca^–/–^* HSCs share increased R-loop and dsRNA levels and a ribosomal biogenesis defect. Further, *Setd2^+/–^ Fanca^–/–^* HSCs displayed cell cycle arrest, mitotic errors, and BMF phenotypes. Importantly, utilizing our *Setd2^+/–^ Fanca^–/–^* mice, we discovered that Juglone, a pan-RNAP inhibitor, reduces R-loop and dsRNA and reverses ribosomal biogenesis defects and mitotic errors, thereby rescuing BMF. This study establishes a mouse model that underscores a key role for R-loop formation, ribosomal biogenesis defects, and mitotic errors in HSCs in driving BMF in FA. We also introduce a potential therapeutic avenue based upon pan-inhibition of RNAPs utilizing Juglone.

## Abstract 2

Using novel FA model, Juglone treatment of HSCs downregulates R-loops and dsRNA, resolves ribosomal defects, mitotic errors and rescues HSC function in FA.

## Graphical abstract

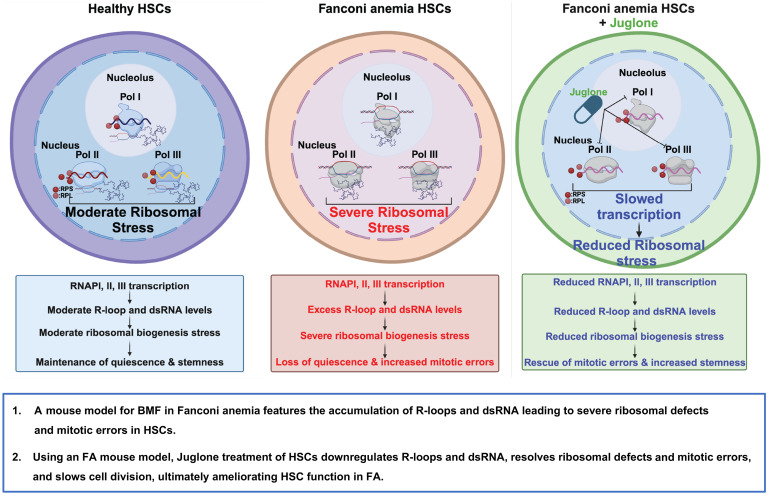

## Introduction

The hematopoietic system is responsible for providing a continuous supply of various mature blood cell types, including erythrocytes, platelets, granulocytes, monocytes, and lymphocytes (1–3). Hematopoiesis depends on hematopoietic stem cells (HSCs), which can either self-renew or differentiate to multi-potent progenitors (1, 2). However, under certain disease conditions, a failure to maintain the HSC pool leads to BM failure (BMF). BMF is characterized by hypoplastic BM and/or peripheral cytopenia in one or more types of blood cells (4, 5). BMF can have serious consequences, including a predisposition to leukemia and an increased rate of death (5, 6). Fanconi anemia (FA) is the most common inherited BMF syndrome (7, 8). As such, understanding the etiology is important for diagnosing and treating BMF in FA. FA is characterized by progressive pancytopenia, as well as variable congenital anomalies and an elevated risk of developing myelodysplastic syndromes, leukemia, and solid tumors (9, 10).

Twenty-three FANC genes that cause FA when mutated have been identified (10–12). These correspond to distinct genetic complementation groups, including FA A–C, D1, D2, E–G, I–J, L–W, and Y. Although *FANCB* is X-linked, FA genes are typically recessive. *FANCA* is by far the most frequently mutated gene in FA, representing approximately 60% of cases (13, 14). There is some phenotypic heterogeneity among complementation groups (10, 15).

The FA nuclear core complex, which includes FANC A, B, C, E, F, G, L, and M, along with FANCT, has a ubiquitin ligase activity that monoubiquitinates FANCD2 and FANCI via a process termed the FA pathway (10, 14, 16–21). Monoubiquitinated FANCD2-FANCI form a DNA clamp (22, 23). Among other FA proteins are those not required for mono-ubiquitination of FANCD2 (or FANCI), including key homologous recombination proteins such as BRCA1/FANCS, BRCA2/FANCD1, PALB2/FANCN, RAD51/FANCR, RAD51C/FANCO, and XRCC2/FANCU (15, 24–30). Other proteins encoded by FA/FA-like genes include the nucleotide excision repair protein XPF/FANCQ, the SLX4/FANCP endonuclease, the REV7/FANCV trans-lesion polymerase, and RFWD3/FANCW (11, 31–34).

The different FA complementation groups display a shared cellular sensitivity and chromosome abnormality phenotype in response to agents such as mitomycin C (MMC) that cause DNA interstrand cross-links (10, 16, 35). It is, therefore, widely accepted that FA proteins have a role in interstrand cross-link repair. More recent work has suggested a broader role for FA proteins in responding to replication stress (12, 36–41). One way by which FA proteins ameliorate replication stress and protect genome stability is by mitigating R-loops, which are RNA-DNA hybrids that displace the complementary DNA strand (42–44). R-loops can arise from the collision of the transcription machinery and the replication fork and can be resolved, in part, by helicases and RNases (45). One place that the resolution of R-loops and transcription-replication conflicts may be particularly critical is in nucleoli, given that rRNA genes are among the most actively transcribed in cells (46).

Importantly, whether R-loops lead to BMF in FA, and if so by what mechanism, has remained elusive. Further, single-gene KO of FA genes, including *Fanca*, in mice does not lead to a clear BMF phenotype. Since RNA polymerase (RNAP) can indirectly generate R-loops by transcribing DNA into RNA, and because SETD2 restricts RNAP-dependent transcriptional elongation (47), we have generated *Fanca* and *Setd2* double mutants in mice as a means to explore the role of R-loops in BMF in FA. In support of such an approach, *SETD2* is essential for hematopoiesis, and disruption of *SETD2* leads to leukemia (47). The SETD2 protein methylates histone H3 at lysine 36 (H3K36me3), thereby ensuring accurate transcription of genes necessary for blood cell development by promoting RNAP-dependent elongation of transcripts. Notably, RNAPs I, II, and III occasionally form R-loops during transcription that can generate dsRNA, which have a variety of transcriptional regulatory functions (46), including gene silencing through RNAi pathways (48). However, excessive accumulation of dsRNA can also lead to transcriptional dysregulation and genomic instability and can thereby contribute to diseases such as cancer and BMF.

RNAPs have specialized roles: RNAPI transcribes rRNA genes, RNAPII transcribes mRNA and certain small RNAs, and RNAPIII transcribes tRNA and the 5S rRNA gene (49). A variety of RNAP inhibitors have been developed that can be utilized to dissect the roles of distinct RNAPs in biological processes, including an RNAPI inhibitor, CX5461 (pidnarulex), which was in an early-stage clinical trial and is effective against tumors with *BRCA1* and *BRCA2* mutations (50). Juglone, 5-hydroxy-1,4-naphthoquinone, inhibits members of the parvulin PPIase family as well as RNAP I, II, and III (and is therefore a pan-RNAP inhibitor) (51). In addition, L-leucine is a branched-chain amino acid that can activate mRNA translation via mTORC1 activation. L-leucine has been used to improve the treatment of Diamond-Blackfan anemia in clinical trials (52).

In this study, we established an FA mouse model (*Setd2^+/–^ Fanca^–/–^*), which displays severe BMF more similar to that observed in patients with FA than in single–FA gene KO mice. Further, we observed excessive R-loop and dsRNA accumulation and ribosomal biogenesis defects in cells from a patient with FA (FA-A complementation group) as well as HSCs from *Setd2^+/–^ Fanca^–/–^* mice. Importantly, given that the pan-RNAP inhibitor Juglone ameliorated the ribosomal biogenesis defect and reduced mitotic errors in *Setd2^+/–^ Fanca^–/–^* HSCs, our results support a key role for R-loops generated at rDNA loci, and potentially for dsRNA and a resulting ribosomopathy, as well as mitotic defects, as a driver of FA-associated BMF. Further, our results suggest that inhibition of RNAPs may provide a therapeutic alternative to BM transplantation to prevent and/or treat FA-related BMF.

## Results

### R-loop and dsRNA accumulation results in ribosomal biogenesis defects in FANCA-deficient cells.

**Figure 1:**
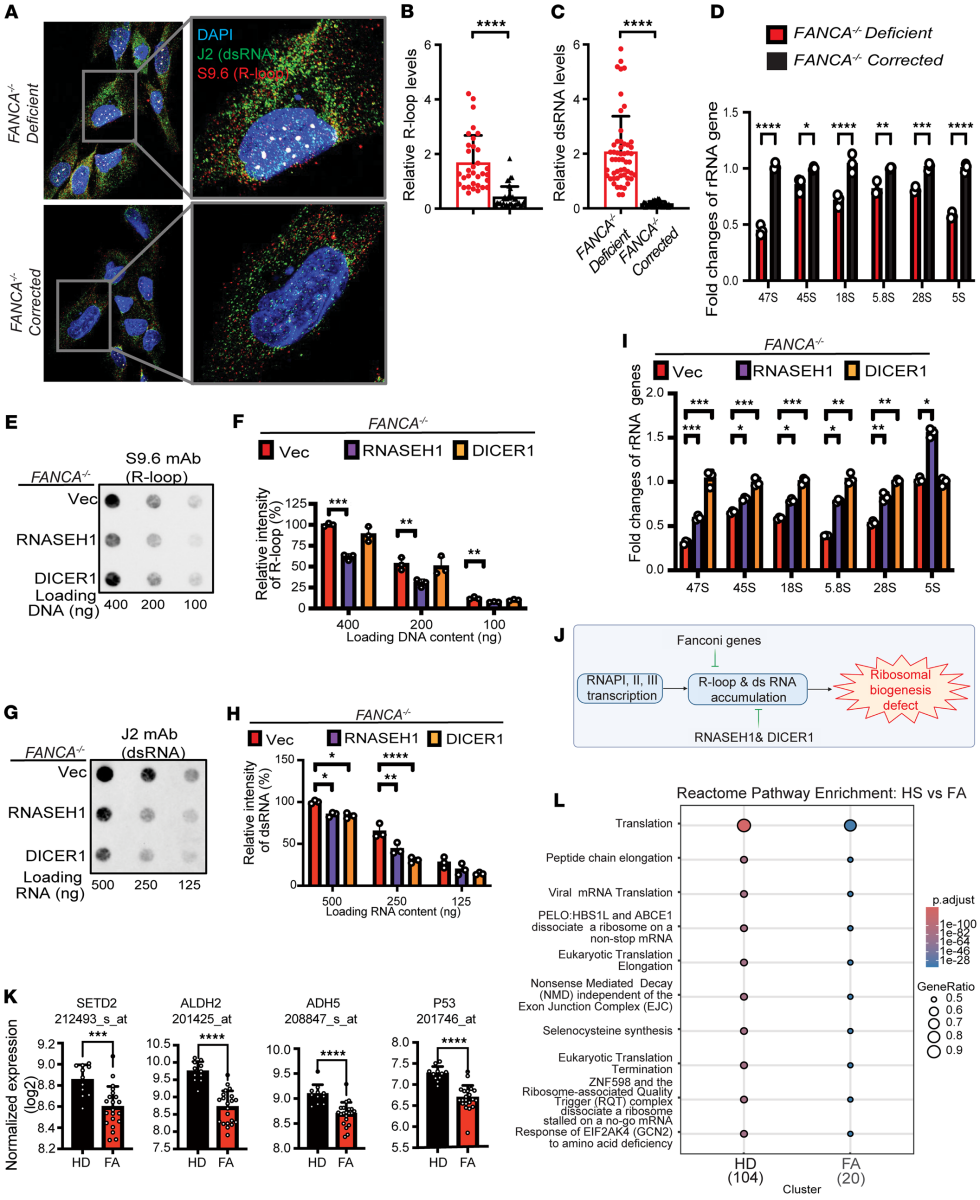
Human *FANCA*-deficient cells from a patient with FA display accumulation of R-loops and dsRNA and errors in ribosomal biogenesis. (**A**) Visualization of R-loops detected by S9.6 mAb (red) and dsRNA detected by rJ2 mAb (green) and via immunofluorescence microscopy in *FANCA*-corrected or -deficient cells from a patient with FA. Nuclei shown by counterstaining with DAPI (blue). Original magnification is ×63. (**B** and **C**) Quantification of R-loops (**B**) and dsRNA (**C**) based upon the area integrated intensity measured by ImageJ (NIH). One-tailed Student’s *t* test; *n* = 37 for each condition. (**D**) Quantification of the expression of ribosomal genes using qRT-PCR. One-tailed Student’s *t* test; *n* = 3 for each condition. (**E**) DNA dot blots detecting R-loop levels using S9.6 antibody in *FANCA*-deficient cells reconstituted with 2 RNases (RNASEH1 or DICER1) or vector alone (Vec). (**F**) Quantification of R-loop levels shown in **E**. One-way ANOVA with Tukey’s multiple-comparison test; *n* = 3 for each condition. (**G**) RNA dot blots detecting levels of dsRNA in *FANCA*-deficient cells reconstituted with 2 RNases (RNASEH1 or DICER1) or Vec. *n* = 3 for each condition. (**H**) Quantification of dsRNA levels shown in **G**. One-way ANOVA with Tukey’s multiple-comparison test; *n* = 3 for each condition. (**I**) Quantification of specific rRNA forms in *FANCA*-deficient cells containing empty vector with or without overexpression of *RNASE H1* or *DICER1*. One-way ANOVA with Tukey’s multiple-comparison test; *n* = 3 for each condition. (**J**) Visual summary. (**K** and **L**) For patient-derived cells, we utilized *FANCA*-deficient GM6935 cells (84). (**K**) Relative expression levels of SETD2, ALDH2, ADH5, and TP53 in healthy donors (*n* = 11) and FA patients (*n* = 21) were analyzed using normalized microarray data (GSE16334). Expression values presented as log_2_-transformed intensities. One-tailed Student’s *t* test. (**L**) Reactome pathway enrichment analysis comparing HD and FA gene clusters performed using compareCluster function (fun = “enrichPathway”, pvalueCutoff = 0.05) from clusterProfiler and ReactomePA. Top 10 enriched pathways are shown in dot plot: color indicates adjusted *P* value; dot size represents gene ratio. *P < 0.05, **P < 0.01, ***P < 0.001, ****P < 0.0001.

Multiple reports have shown accumulation of R-loops in FA mutant cells (42-44). Thus, to better understand cellular defects associated with FA, we initially assessed the levels of R-loops in corrected (*FANCA* corrected) and uncorrected (*FANCA* deficient) pairs of FA patient–derived fibroblasts. Indeed, R-loops were expressed at higher levels in *FANCA*-deficient cells compared with *FANCA*-corrected cells (Figure 1, A and B). Additionally, when R-loops are formed in certain genes, they can trigger the production of another type of RNA, dsRNA (46). Strikingly, *FANCA*-deficient cells also had higher levels of dsRNA than *FANCA*-corrected cells (Figure 1, A and C). Given that R-loops were more abundant in *FANCA*-deficient cells, and since RNAPI-dependent rRNAs are among the most active sites of transcription in the cell (43, 53), we hypothesized that rRNAs might be compromised by deficiency for *FANCA*. As anticipated, all rRNAs transcribed in nucleoli, as well as RNAPIII-dependent 5S, were downregulated in patient-derived *FANCA-*deficient fibroblasts (Figure 1D).

To address the role of R-loops and dsRNA in regulating transcription within nucleoli, we conducted ectopic expression experiments of RNASEH1 and DICER1 in *FANCA*-deficient cells (Figure 1, E–I). The expression of RNASEH1 led to a significant reduction in both R-loop and dsRNA levels, while DICER1 expression specifically resolved dsRNA in *FANCA*-deficient cells (Figure 1, E–H). Importantly, ectopic expression of both RNASEH1 and DICER1 resulted in upregulated expression of various rRNAs (Figure 1I). Taken together, these results suggest that resolving the accumulation of R-loops and, somewhat surprisingly dsRNA, may rescue ribosomal biogenesis defects associated with FA (Figure 1J). In support of this, levels of mono-ribosomes and polysomes were indeed decreased in FA patient cells (Supplemental Figure 1; supplemental material available online with this article; https://doi.org/10.1172/jci.insight.192126DS1). Additionally, we analyzed microarray data from low-density BM cells derived from patients with FA (GSE16334). This analysis revealed that, in addition to genes previously implicated in FA-associated BMF mouse models, such as *ALDH2* and *ADH5* (54), *SETD2* expression was also significantly downregulated in FA patient samples (Figure 1K and Supplemental Figure 2), as were genes related to translation/ribosome biogenesis (Figure 1L).

### Development of an FA mouse model with BMF.

**Figure 2:**
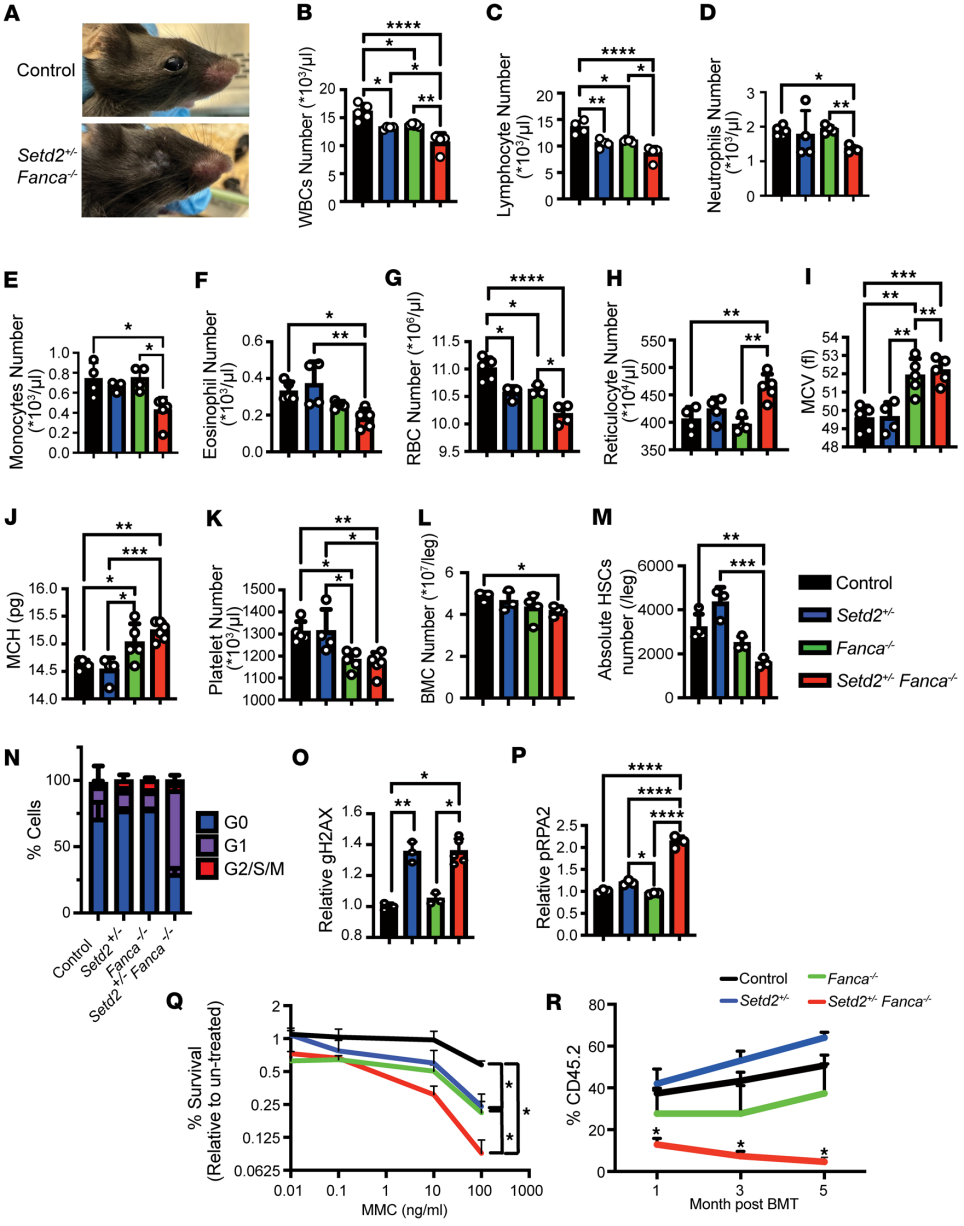
Characterization of BMF in a mouse model for FA. (**A**) Representative photos of eyes in control and *Setd2^+/–^ Fanca^–/–^* mice. (**B**–**K**) Peripheral blood (PB) analysis between control (black), *Setd2^+/–^* (blue), *Fanca^–/–^*(green), and *Setd2^+/–^ Fanca^–/–^* (red) mice. WBCs (**B**), neutrophils (**D**), monocytes (**E**), eosinophils (**F**), RBCs (**G**), reticulocytes (**H**), MCV (**I**), MCH (**J**), platelets (**K**). One-way ANOVA with Tukey’s multiple-comparison test. (**L**) Total BM cell number in control (black, *n* = 3), *Setd2^+/–^* (blue, *n* = 3), *Fanca^–/–^* (green, *n* = 4), and *Setd2^+/–^ Fanca^–/–^* (red, *n* = 4) mice. One-way ANOVA with Tukey’s multiple-comparison test. (**M**) Absolute HSC [lineage (–), c-Kit (+), EPCR (+), CD48 (–), and CD150 (+)] numbers for control (black, *n* =4), *Setd2^+/–^* (blue, *n* = 3), *Fanca^–/–^* (green, *n* = 3), and *Setd2^+/–^ Fanca^–/–^* (red, *n* = 3) mice. One-way ANOVA with Tukey’s multiple-comparison test. (**N**) Cell cycle status by flow cytometry utilizing Ki67 to distinguish G0 cells from G1/S/G2/M cells with DAPI stain to measure DNA content. Graphs show distribution of cells to indicated cell cycle phases. One-way ANOVA with Tukey’s multiple-comparison test; *n* = 4 for each condition. (**O**) Levels of DNA damage in HSCs from control (black, *n* = 4), *Setd2^+/–^* (blue, *n* = 3), *Fanca^–/–^* (green, *n* = 3), and *Setd2^+/–^ Fanca^–/–^* (red, *n* = 5) mice as measured by γH2A.X levels. One-way ANOVA with Tukey’s multiple-comparison test. (**P**) Levels of pRPA2 in HSC from control (black, *n* = 3), *Setd2^+/–^* (blue, *n* = 3), *Fanca^–/–^* (green, *n* = 3), and *Setd2^+/–^ Fanca^–/–^* (red, *n* = 3) mice. One-way ANOVA with Tukey’s multiple-comparison test. (**Q**) MMC sensitivity for c-Kit^+^ cells from control (black), *Setd2^+/–^* (blue), *Fanca^–/–^* (green), and *Setd2^+/–^ Fanca^–/–^* (red) mice. c-Kit^+^ cells were cultured with MMC for 3 days when cell numbers were counted using Celigo. Two-way ANOVA with Tukey’s multiple-comparison test; *n* = 4 for each condition. (**R**) BM transplantation of CD45.2^+^ control (black), *Setd2*^+/–^ (blue), *Fanca^–/–^* (green), and *Setd2^+/–^ Fanca^–/–^* (red) BMCs into CD45.1 mice. After recipient mice received lethal irradiation, BMCs from control, *Setd2^+/–^*, *Fanca^–/–^*, and *Setd2^+/–^* *Fanca^–/–^* were injected into recipient mice. PB was analyzed at 1, 3, and 5 months after transplantation. Two-way ANOVA followed by Tukey’s multiple-comparison test; *n* = 5 for each condition. Data shown as mean ± SEM. *P < 0.05, **P < 0.01, ***P < 0.001, ****P < 0.0001.

Single-KO mouse models of FA, such as the *Fanca*-KO model, do not fully recapitulate the human disease including an absence of BMF (55). However, since FA patient–derived fibroblasts displayed accumulation of R-loops and dsRNA, as well as ribosomal biogenesis defects, we hypothesized that enhancing RNAP-dependent elongation in mice might produce a BMF phenotype. Importantly, *SETD2* restricts RNAPs (47), and SETD2 was downregulated in FA patient–derived low-density BM cells (Figure 1K). Thus, we generated a compound mutant mouse model: *Setd2^+/–^ Fanca^–/–^*. Furthermore, we demonstrated that the *Setd2^–/–^* mutant is embryonic lethal in our previous report (47). Indeed, *Setd2^+/–^ Fanca^–/–^* mice exhibited phenotypes reminiscent of those seen in patients with FA, including defects of eyelids and eyeballs (Figure 2A). Importantly, as compared with *Setd2^+/–^* and/or *Fanca^–/–^* mice, *Setd2^+/–^ Fanca^–/–^* mice exhibited further reductions in the total numbers of WBCs, neutrophils, monocytes, eosinophils, RBCs, platelets, BM cells, and HSCs and increased reticulocytes, mean corpuscular volume (MCV), and mean corpuscular hemoglobin (MCH) (Figure 2, B–M). For further insight into the effects of the combination of *Setd2^+/–^* and *Fanca^–/–^* mutations on HSC function, we evaluated the cell cycle status of HSCs from adult-stage mice. As compared with HSCs from control mice, those from *Setd2^+/–^ Fanca^–/–^* mice, but not *Setd2^+/–^* or *Fanca^–/–^* single-mutant mice, displayed a decrease in quiescent (G0) cells accompanied by an increase in G1 cells (Figure 2N). Interestingly, while *Fanca^–/–^* single-mutant HSCs had elevated DNA damage compared with WT HSCs, as measured by relative levels of γH2A.X, DNA damage was further elevated in *Setd2^+/–^ Fanca^–/–^* HSCs to similar levels as in *Setd2^+/–^* single mutants (Figure 2O). As a measure of replication stress (56), in addition to γH2AX, pRPA2, pCHK1, and pCHK2 were significantly upregulated in *Setd2^+/–^ Fanca^–/–^* HSCs (Figure 2, O and P, and Supplemental Figure 3). In this context, it should be noted that hypersensitivity to DNA cross-linking agents, such as MMC, is a well-known characteristic of FA cells, including FA-A cells deficient for *FANCA* (57), and HSCs from FA single-gene KO mice (58). An examination of the MMC sensitivity of c-Kit–positive cells further supports the conclusion that the combination of the *Setd2^+/–^* and *Fanca^–/–^* mutations enhances FA phenotypes. Cells from *Setd2^+/–^* and *Fanca^–/–^* single-mutant mice exhibited increased sensitivity to MMC, as compared with WT control cells, and this was enhanced even further in cells from double-mutant *Setd2^+/–^ Fanca^–/–^* mice (Figure 2Q). To further analyze HSC function in *Setd2^+/–^ Fanca^–/–^* mice, we performed a competitive BM transplantation assay. The results indicate that, as compared with *Setd2^+/–^* or *Fanca^–/–^* mouse-derived BM cells (BMCs), *Setd2^+/–^ Fanca^–/–^* mouse–derived BMCs exhibited a dramatic loss of engraftment capacity from the first month after BM transplantation (Figure 2R). Taken together, these findings suggest that the combination of *Setd2^+/–^* and *Fanca^–/–^* mutations results in more pronounced DNA damage and defects in HSC maintenance, leading to BMF.

**Figure 3:**
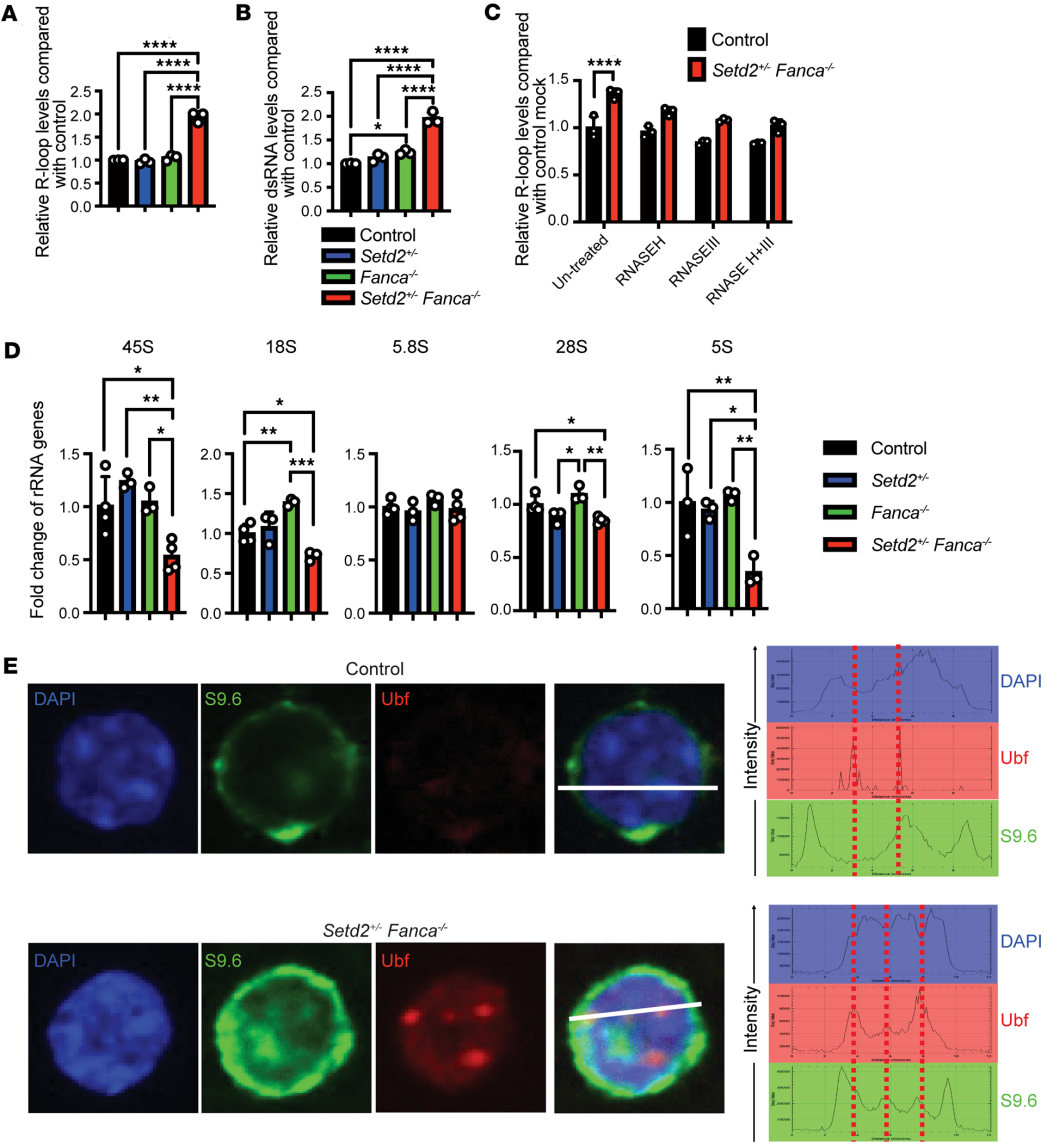
R-loop accumulation and ribosomal biogenesis defect in *Setd2^+/–^ Fanca^–/–^* mouse model HSCs. (**A**) Levels of R-loop in HSC from control (black, *n* = 3), *Setd2^+/–^* (blue, *n* = 3), *Fanca^–/–^* (green, *n* = 3), and *Setd2^+/–^ Fanca^–/–^* (red, *n* = 3) mice. One-way ANOVA with Tukey’s multiple-comparison test. (**B**) Levels of dsRNA in HSC from control (black, *n* = 3), *Setd2^+/–^* (blue, *n* = 3), *Fanca^–/–^* (green, *n* = 3), and *Setd2^+/–^ Fanca^–/–^* (red, *n* = 3) mice. One-way ANOVA with Tukey’s multiple-comparison test. (**C**) Levels of R-loops in HSCs from control and *Setd2^+/–^ Fanca^–/–^* mice, with or without treatment of various RNases, were analyzed by flow cytometry using S9.6 antibody. One-tailed Student’s *t* test; *n* = 3 for each condition. (**D**) Ribosomal gene expression in control, *Setd2^+/–^*, *Fanca^–/–^*, and *Setd2^+/–^ Fanca^–/–^* HSCs measured utilizing qRT-PCR. Control (black, *n* = 4), *Setd2^+/–^* (blue, *n* = 3), *Fanca^–/–^* (green, *n* = 3), and *Setd2^+/–^ Fanca^–/–^* (red, *n* = 4). One-way ANOVA with Tukey’s multiple-comparison test. (**E**) Measures of colocalization of R-loops (green, detected using S9.6 antibody) with RNA polymerase I (red, detected using Ubf antibody); DNA is counterstained with DAPI (blue). Data are shown as mean ± SEM. *P < 0.05, **P < 0.01, ***P < 0.001, ****P < 0.0001.

Next, we investigated whether BMF and other FA-related phenotypes of HSCs in our *Setd2^+/–^ Fanca^–/–^* mouse model might be associated with dysregulation of R-loops and rRNA levels since these were seen in *FANCA*-deficient fibroblasts (Figure 1). Indeed, similar to patient-derived FA-A cells, *Setd2^+/–^ Fanca^–/–^* mice showed accumulation of R-loops and dsRNA that were elevated in comparison to HSCs from WT controls and *Setd2^+/–^* and *Fanca^–/–^* single mutants (Figure 3, A and B). Importantly, R-loop levels could be reduced in HSCs from *Setd2^+/–^ Fanca^–/–^* mice via expression of various RNases (Figure 3C). Further, most rRNAs, such as 5S, 18S, 28S, and 45S, were significantly downregulated in *Setd2^+/–^ Fanca^–/–^* mouse HSCs as compared with the WT or each single mutant (Figure 3D). Additionally, an immunofluorescent imaging analysis showed that the upstream binding factor, a marker for ribosomal loci (59), mostly colocalized with R-loops at nucleoli in *Setd2^+/–^ Fanca^–/–^* HSCs (Figure 3E and Supplemental Figure 4A). Since upstream binding factor is related to RNAP I (60), and RNAP III localizes to nucleoli (61), accumulation of R-loops at nucleoli and resulting ribosome biogenesis defects in *Setd2^+/–^ Fanca^–/–^* HSCs may be related to RNAPI and maybe also RNAPIII. Further, because R-loops accumulated at ribosomal loci, and there was a defect in various rRNA levels (Figure 3E), similar to that observed in cells from patients with FA, our *Setd2^+/–^ Fanca^–/–^* double-mutant mice may serve as a model for FA-associated BMF driven by ribosomal biogenesis defects.

### Mitotic errors in Setd2^+/–^ Fanca^–/–^ HSCs.

**Figure 4:**
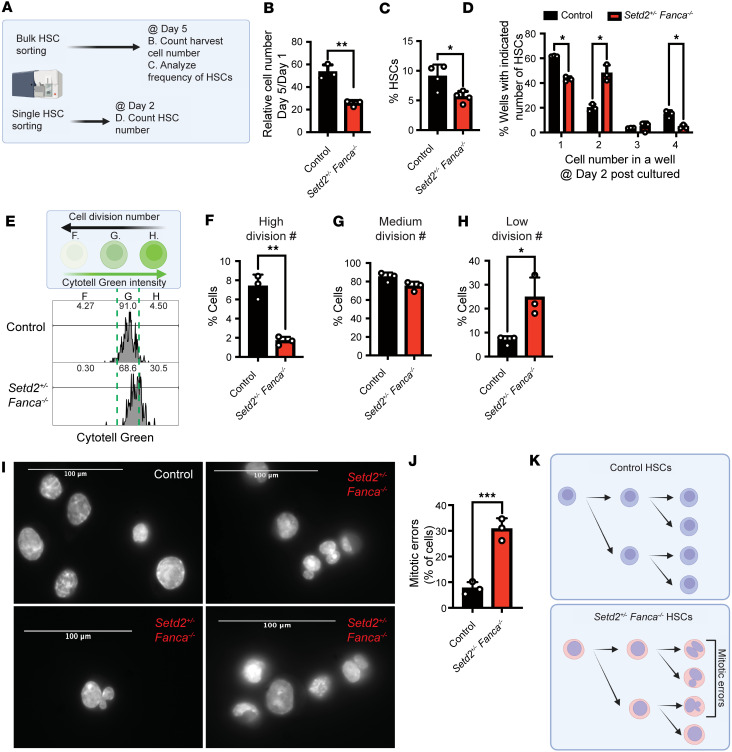
HSCs from *Setd2^+/–^ Fanca^–/–^* mice display cell division defects. (**A**) Schematic of the experimental setup for **B**–**D**. (**B** and **C**) Proliferative capacity (**B**) and HSC [lineage (–), c-Kit (+), EPCR (+), CD48 (–), and CD150 (+)] frequency (**C**) of control (WT) or *Setd2^+/–^ Fanca^–/–^* HSCs, determined as number of cells after 5 days of liquid culture relative to number at day 1, and HSCs as percentage of total at day 5, respectively. Cultures of HSCs were counted with Celigo in **B** and were analyzed by flow cytometry in **C**; for each condition, *n* = 3 in **B** and *n* = 4 in **C**. One-tailed Student’s *t* test. (**D**) Counts by hand of the number of cells in each well at 2 days after establishing single-cell cultures of control or *Setd2^+/–^ Fanca^–/–^* HSCs. One-tailed Student’s *t* test; *n* = 3 for each condition. (**E**–**H**) Analysis comparing the cell division histories of control versus *Setd2^+/–^ Fanca^–/–^* HSCs in 5-day liquid cultures. HSCs were sorted and stained with CytoTell green, then cultured for 5 days. At day 5, HSCs were stained with surface markers, and cell division history was analyzed with flow cytometry based on the CytoTell green signal in various peaks. Low division, medium division, and high division numbers were based on CytoTell green signal associated with 1–3, 4–8, and 9 or more divisions, respectively. One-tailed Student’s *t* test; *n* = 3 for each condition. (**I**) Visualization of nuclei in HSCs from control and *Setd2^+/–^ Fanca^–/–^* mice, as a measure of mitotic errors, after 5 days in liquid culture. (**J**) Quantification of cells with abnormal nuclei (binucleated or micronucleated), indicating mitotic errors. One-tailed Student’s *t* test; *n* = 3 for each condition. (**K**) Schematic depicting nuclear morphologies and mitotic errors in controls and *Setd2^+/–^ Fanca^–/–^* HSCs during cell proliferation. Data are shown as mean ± SEM. *P < 0.05, **P < 0.01, ***P < 0.001.

Since a failure to enter the quiescent-G0 phase may cause BMF in our *Setd2^+/–^ Fanca^–/–^* mouse model (Figure 2N), and cell division defects have been reported in fibroblasts deficient for FA factors including FANCA (62), we conducted liquid culture experiments of HSCs to carefully examine their cell division process (Figure 4A). Notably, as compared with the control, *Setd2^+/–^ Fanca^–/–^* double-mutant HSCs exhibited diminished proliferation seen as a decreased HSC population (Figure 4, B and C). Additionally, we analyzed the cell division history via single-cell culture (Figure 4D) and CytoTell green–based cell division history assay (Figure 4, E–H) with sorted HSCs from control or *Setd2^+/–^ Fanca^–/–^* mice. This experiment indicates that almost 40% of *Setd2^+/–^ Fanca^–/–^* HSCs underwent a first cell division within 2 days, whereas only 20% of control HSCs did so (Figure 4D). However, an examination of 5-day cultures revealed that, in comparison to the control, *Setd2^+/–^ Fanca^–/–^* HSCs showed an arrest during their first cell division and failed to complete successive cell divisions (Figure 4, E–H). To clarify the basis for the arrested cell division in *Setd2^+/–^ Fanca^–/–^* HSCs, we examined nuclear morphologies utilizing immunofluorescence microscopy. There was an increased number of abnormal binucleated and micronucleated cells in *Setd2^+/–^ Fanca^–/–^* HSCs (Figure 4, I and J). These results suggest that mitotic errors underlie defects in *Setd2^+/–^ Fanca^–/–^* HSC function, including decreased proliferation (Figure 2N and Figure 4, E–K) and decreased chimerism in BM transplants (Figure 2R).

### Inhibition of RNAPs rescues ribosomal biogenesis defects and survival of HSCs in Setd2^+/–^ Fanca^–/–^ mice.

**Figure 5:**
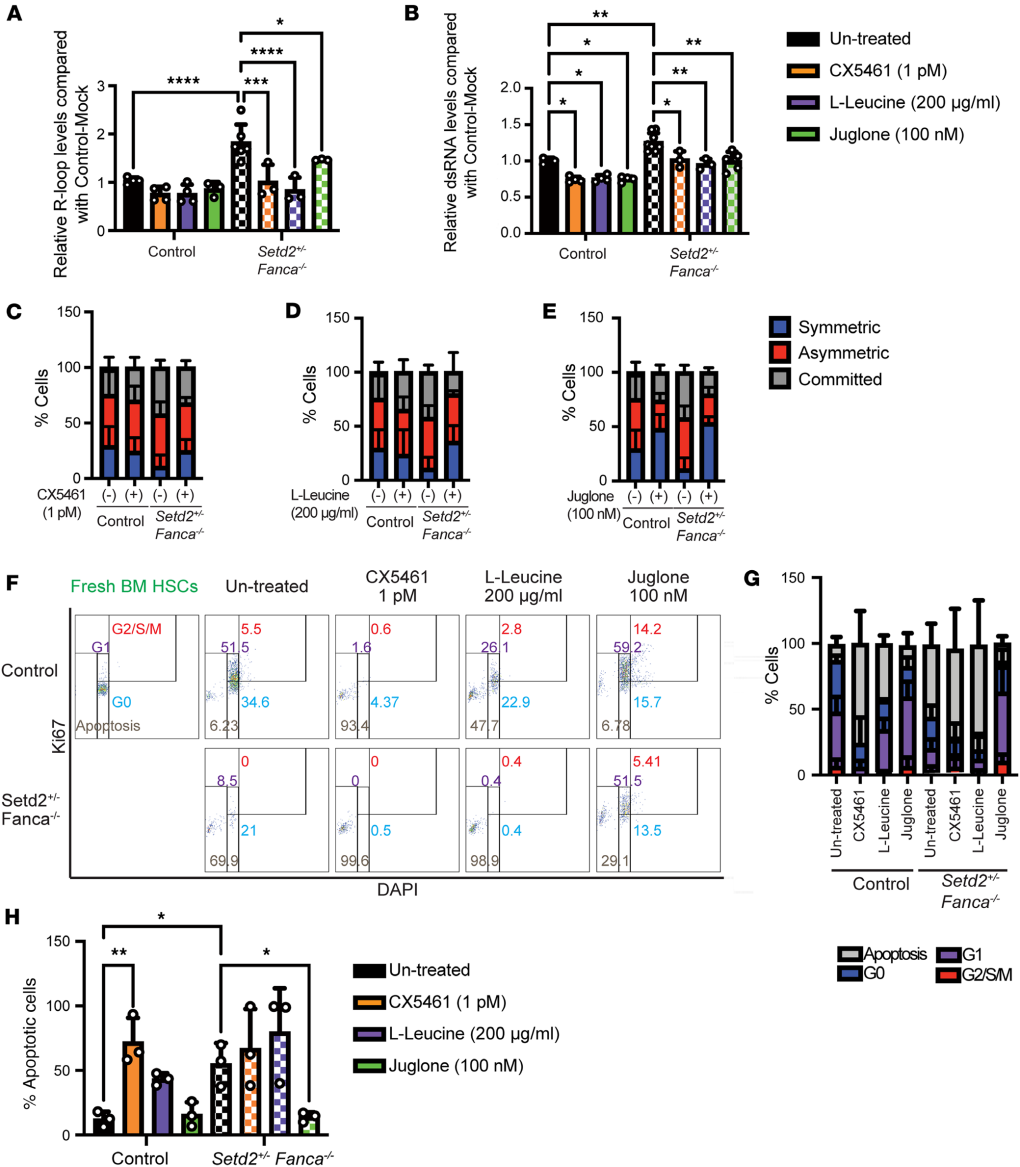
Inhibition of RNA polymerases reduces R-loops and cell cycle defects and rescues self-renewal capacity and BMF of *Setd2^+/–^ Fanca^–/–^* HSCs. (**A** and **B**) The levels of S9.6 (R-loops) in untreated (black, control: *n* = 4, *Setd2^+/–^ Fanca^–/–^*: *n* = 6) versus CX5461 (orange, control: *n* = 4, *Setd2^+/–^ Fanca^–/–^*: *n* = 3), L-leucine (purple, control: *n* = 4, *Setd2^+/–^ Fanca^–/–^*: *n* = 3), and Juglone-treated (green, control: *n* = 3, *Setd2^+/–^ Fanca^–/–^*: *n* = 3) HSPCs (**A**) and J2 (dsRNA) in untreated (black, control: *n* = 3, *Setd2^+/–^ Fanca^–/–^*: *n* =7) versus CX5461 (orange, control: *n* =4, *Setd2^+/–^ Fanca^–/–^*: *n* = 3), L-leucine (purple, control: *n* = 4, *Setd2^+/–^ Fanca^–/–^*: *n* = 3), and Juglone-treated (green, control: *n* = 4, *Setd2^+/–^ Fanca^–/–^*: *n* = 5) HSPCs (**B**). One-way ANOVA with Tukey’s multiple-comparison test. (**C**–**E**) Paired-daughter assay with or without CX5461(**C**), L-leucine (**D**), and Juglone (**E**). One-way ANOVA with Tukey’s multiple-comparison test. (**F**–**H**) Cell cycle and apoptotic effects of CX5461, L-leucine, and Juglone treatment of control or *Setd2^+/–^ Fanca^–/–^* HSCs are shown. (**F**) Ki67 staining after Juglone treatment as shown in FACS plots. (**G**) Frequency of apoptosis and of each cell cycle stage in HSCs. One-way ANOVA with Tukey’s multiple-comparison test; *n* = 3 for each condition. (**H**) Frequency of apoptotic or pre-apoptotic cells. One-way ANOVA, Tukey’s multiple-comparison test; *n* = 3 each condition. Data are shown as mean ± SEM. *P < 0.05, **P < 0.01, ***P < 0.001, ****P < 0.0001.

One approach to translating our findings of excessive cycling of *Setd2^+/–^ Fanca^–/–^* HSCs, ultimately leading to mitotic errors and BMF into a potential therapy for BMF in patients with FA, is to reduce R-loop accumulation. Ideally, a drug treatment to reduce R-loop accumulation would suppress DNA damage and/or rescue HSC mitotic defects. Thus, for this purpose, we employed a stimulator of mRNA translation (L-leucine) (52), an RNAP I inhibitor (CX5461) (50), and a pan-RNAP inhibitor (Juglone) (51) in liquid cultures of isolated HSCs. Given that FA cells exhibit ribosomal defects (Supplemental Figure 1), activating mRNA translation through L-leucine, or inhibiting transcription and thus R-loop generation, may ameliorate HSC function. To validate these hypotheses, we treated *Setd2^+/–^ Fanca^–/–^* hematopoietic stem and progenitor cells (HSPCs) in liquid culture with 200 μM of L-leucine, 1 pM of CX5461, or 0.1 μM of Juglone and measured R-loop (S9.6) and dsRNA (J2) levels. CX5461, L-leucine, and Juglone treatment reduced the levels of both R-loops and dsRNA (Figure 5, A and B). As a measure of whether this reduction in R-loop levels aids HSC function, we conducted single cell–based paired-daughter assays. Symmetric division maintains stemness, while asymmetric division leads to 1 HSC and 1 cell committed to division that eventually generates 4 myeloid lineages (47). Treatment with L-leucine and CX5461, and especially Juglone, increased symmetric cell divisions and decreased committed asymmetric cell divisions of *Setd2^+/–^ Fanca^–/–^* HSCs (Figure 5, C–E). This suggests that low doses of L-leucine, CX5461, and Juglone help maintain the stemness of *Setd2^+/–^ Fanca^–/–^* HSCs. Interestingly, Juglone treatment also improved symmetric division in control HSCs.

To further analyze the mechanism of how L-leucine, CX5461, and Juglone facilitate the maintenance of *Setd2^+/–^ Fanca^–/–^* HSC stemness, we performed cell cycle assays with treated cells. Unlike CX5461 and L-leucine, Juglone rescued survival of *Setd2^+/–^ Fanca^–/–^* HSCs by reducing the number of apoptotic cells and restored levels of G0 and G1 cells to those similar to untreated controls (Figure 5, F–H). These results suggest that Juglone may have the potential to reverse BMF, as Juglone treatment was found to maintain HSC survival from *Setd2^+/–^ Fanca^–/–^* mice.

### The pan-RNAP inhibitor Juglone slows cell division, which ameliorates the maintenance of stemness in Setd2^+/–^ Fanca^–/–^ HSCs.

**Figure 6:**
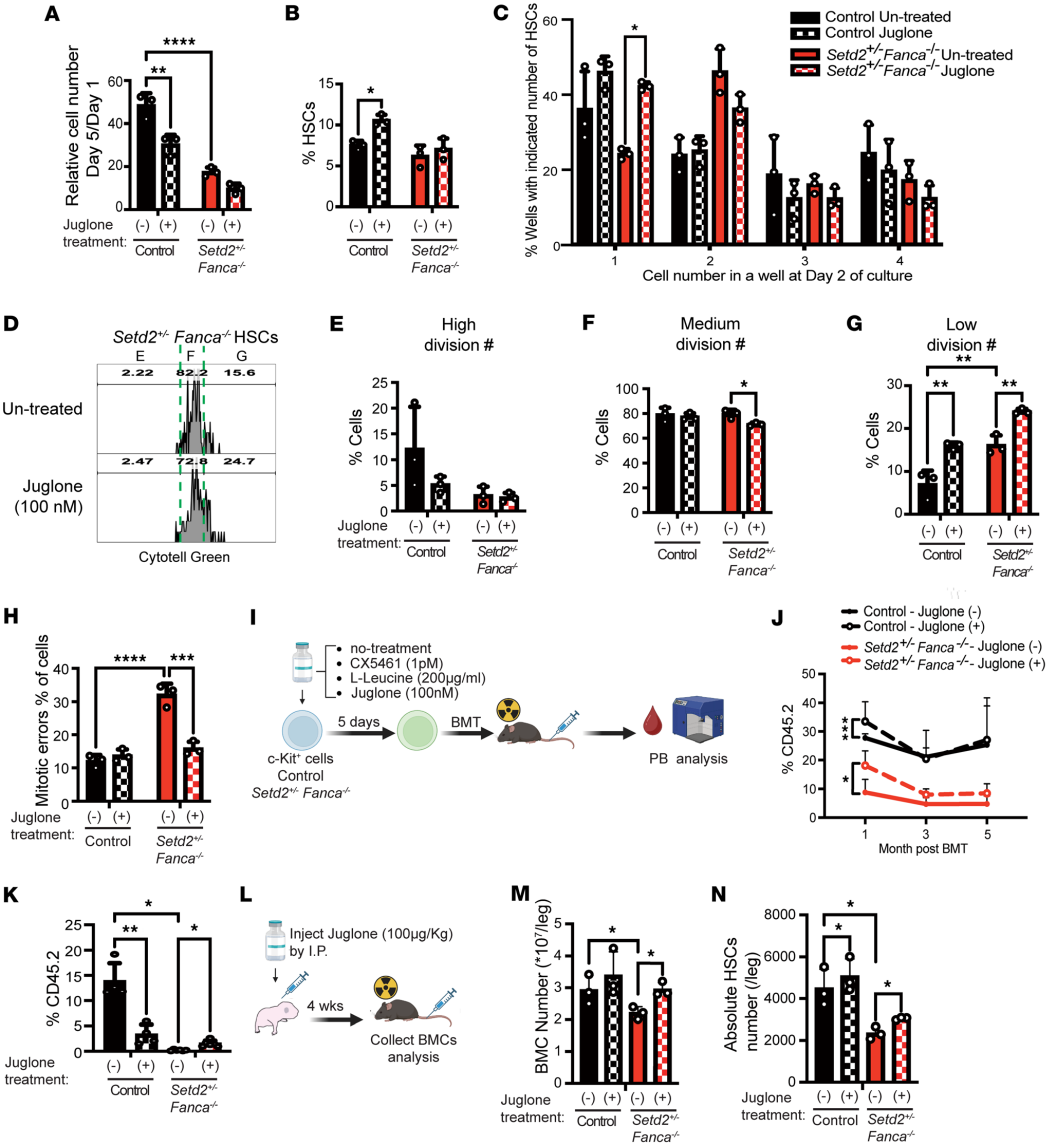
Treatment with Juglone slows cell division and facilitates the maintenance of stemness and improves engraftment of *Setd2^+/–^ Fanca^–/–^* HSCs. (**A** and **B**) Proliferation capacity counted by Celigo (**A**) and HSC frequency analyzed by flow cytometry (**B**) of WT control or *Setd2^+/–^ Fanca^–/–^* HSCs after 5 days of liquid culture. *N* = 3 each condition. (**C**) Single-cell culture of control or *Setd2^+/–^ Fanca^–/–^* cells with or without Juglone (0.1 µM) for 2 days. Cell numbers per well counted at day 2. *N* = 3 for each condition. (**D**–**G**) Cell division history analysis of control and *Setd2^+/–^ Fanca^–/–^* HSCs with or without Juglone (0.1 µM). HSCs were sorted, labeled with CytoTell Green, and cultured for 5 days before analysis by flow cytometry. Representative CytoTell plots (**D**) and quantification of high (**E**), medium (**F**), and low (**G**) division histories are shown. *N* = 3 for each condition. (**H**) Mitotic errors (binucleated or micronucleated cells) in *Setd2^+/–^ Fanca^–/–^* HSCs after 5 days of Juglone treatment (0.1 µM) in liquid culture. *N* = 3 for each condition. (**I**) Experimental scheme of BM transplantation with drug-treated HSPCs. (**J**) BM transplantation of control (black) and *Setd2^+/–^ Fanca^–/–^* (red) HSCs, with or without Juglone treatment. Engraftment was measured as the percentage of CD45.2^+^ PB cells at 4, 8, and 12 weeks. *N* = 5 for each condition. (**K**) Secondary transplantation of control (black) and *Setd2^+/–^ Fanca^–/–^* (red) BMCs, with or without Juglone treatment; engraftment measured as CD45.2^+^ PB cells at 10 weeks. *N* = 4 for each condition. (**L**) Experimental scheme of Juglone in vivo treatment. (**M** and **N**) Total BMC numbers (**M**) and absolute HSC numbers (**N**) in control (black) and *Setd2^+/–^ Fanca^–/–^* (red) mice with or without Juglone treatment. *N* = 3 for each point. Data are shown as mean ± SEM. Statistical significance was determined by 1-way ANOVA with Tukey’s multiple-comparison test unless otherwise indicated; panel **J** was analyzed by 2-way ANOVA with Tukey’s multiple-comparison test. *P < 0.05, **P < 0.01, ***P < 0.001, ****P < 0.0001.

To evaluate the effects of Juglone on the maintenance of HSCs, *Setd2^+/–^ Fanca^–/–^* HSCs were cultured in vitro with Juglone. This resulted in reduced proliferation of both control and *Setd2^+/–^ Fanca^–/–^* cells (Figure 6A) and significantly increased the frequency of control HSCs; there was a trend toward upregulation of the frequency of *Setd2^+/–^ Fanca^–/–^* HSCs (Figure 6B). To further validate effects of Juglone on the rate of cell division, a single cell–based cell division assay was performed, which showed that Juglone treatment slowed the first cell division (Figure 6C). We then performed a CytoTell-based cell division history assay, which indicated that Juglone treatment reduced the number of *Setd2^+/–^ Fanca^–/–^* cells with a medium division history and increased the number of both control and *Setd2^+/–^ Fanca^–/–^* cells with a low division history (Figure 6, E–G). These results are consistent with Juglone treatment slowing cell division and allowing HSCs sufficient time to resolve R-loops and/or dsRNA, and thereby preparing for the next cell division. To determine whether Juglone might improve the proliferation of *Setd2^+/–^ Fanca^–/–^* HSCs by reducing mitotic errors, liquid cultures were performed. Indeed, while Juglone treatment did not alter levels of mitotic errors in control HSCs, it significantly reduced mitotic errors by *Setd2^+/–^ Fanca^–/–^* HSCs (Figure 6H). Finally, to affirm the hypothesis that R-loop accumulation contributes to BMF in FA, we performed a BM transplantation assay using treated or untreated control or *Setd2^+/–^ Fanca^–/–^* c-Kit–positive cells (Figure 6, I and J). Notably, c-Kit–positive *Setd2^+/–^ Fanca^–/–^* cells and not controls, treated with Juglone but not CX5461 or L-leucine, showed significantly higher engraftment (Figure 6J and Supplemental Figure 5B). Importantly, Juglone treatment enhanced long-term engraftment capacity for cells from *Setd2^+/–^ Fanca^–/–^* mice, as confirmed by secondary BM transplant (Figure 6K). In addition to the in vitro Juglone treatment experiments, we performed in vivo Juglone treatments. Juglone was administered as a single i.p. injection to P5 neonatal mice. Four weeks after injection, we analyzed total BM cell numbers and HSC numbers. This analysis revealed that Juglone treatment significantly rescued both total BM cellularity and HSC numbers in WT controls and *Setd2^+/–^ Fanca^–/–^* mice (Figure 6, M and N).

Taken together, these results suggest that Juglone treatment slows cell division and gives HSCs enough time to resolve R-loops and dsRNA, and thereby suppress mitotic errors and rescue *Setd2^+/–^ Fanca^–/–^* HSC stemness and engraftment capacity.

## Discussion

In this study, we began by demonstrating that an FA patient–derived cell line accumulates R-loops, as well as dsRNA, and has reduced expression of all rRNA genes, which underlies ribosomal biogenesis defects. R-loop and/or dsRNA accumulation, and rRNA expression, can be ameliorated by overexpression of either RNASEH1 or DICER1. Because single-gene KO for FA genes in mice does not display BMF (55), to explore the relevance of our observations to this important manifestation of FA, we built an FA mouse model based upon *Setd2^+/–^ Fanca^–/–^* double mutants. Indeed, these mice displayed decreased BM cellularity and a greatly reduced function of HSCs in competitive BM transplants (Figure 2). Further, as compared with control (WT) mice and *Setd2^+/–^* and *Fanca^–/–^* single mutants, *Setd2^+/–^ Fanca^–/–^* double-mutant HSCs displayed accumulation of R-loops and dsRNA. Correspondingly, *Setd2^+/–^ Fanca^–/–^* double-mutant HSCs showed ribosomal biogenesis defects that could be rescued by treatment with the pan-RNAP inhibitor Juglone. Additionally, Juglone could rescue key features of *Setd2^+/–^ Fanca^–/–^* HSCs including mitotic errors, decreased quiescence, and a reduced capacity for successive cell divisions, elevated levels of apoptosis, and ultimately, engraftment defects. Taken together, given that *Setd2^+/–^ Fanca^–/–^* HSCs also have elevated levels of DNA damage and increased sensitivity to MMC, these results suggest the importance of R-loops and dsRNA as well as rDNA loci and ribosomal biogenesis defects in HSCs to BMF associated with FA. Notably, this work may have also identified a putative therapeutic option for treating BMF in patients with FA.

In FA research, a large body of work has focused on DNA damage, MMC sensitivity, and HSC transplantation as a basis for understanding the etiology of BMF and/or a predisposition to various malignancies that are associated with this disease (63). Additionally, more recent work has demonstrated that FA cells have increased levels of R-loops and decreased transcription of rRNA genes (64). However, the current study is the first, to our knowledge, to demonstrate increased levels of R-loops and dsRNA and decreased transcription of rRNA genes, which are largely nucleolar, in FA-related HSCs. In particular, we also reveal a role for RNAP-mediated transcription in this process. Further, based on results obtained utilizing our mouse model, we propose that elevated R-loops and dsRNA, including at rDNA loci, lead to increased levels of DNA damage and MMC sensitivity in HSCs, at least in part via transcription-replication conflicts. Thus, our study provides important insights for understanding FA and the BMF that is frequently associated with it.

One key limitation for the FA field is that *Fanca^–/–^* and other single FA gene KO mouse models do not exhibit BMF-like phenotypes (55). Several double-mutant mouse models, such as *Aldh2^–/–^ Fancd2^–/–^* and *Adh5^–/–^ Fancd2^–/–^*, can induce severe BMF (54, 65), which suggests the importance of DNA damage in this process; while *ADH5* directly detoxifies formaldehyde in the nucleus generated by various biochemical pathways, *ALDH2* detoxifies acetaldehyde (a byproduct of alcohol metabolism). However, the role of R-loops and dsRNA in FA-related BMF was previously untested, to our knowledge; our *Setd2^+/–^ Fanca^–/–^* double-mutant mouse model demonstrates the role of these factors in the etiology of BMF in FA and, additionally, may position R-loops and dsRNA at rDNA loci as an important upstream source of DNA damage that also contributes to BMF in FA. The *Setd2^+/–^ Fanca^–/–^* FA model we have built accurately recapitulates cellular defects associated with patients with FA, including increased R-loops and dsRNA, as well as decreased transcription of rDNA and increased sensitivity to MMC (Figures 1–3). As such, this provides a relevant model for studying the disease mechanisms in, and potential treatments for, BMF in FA. The *Setd2^+/–^ Fanca^–/–^* FA mouse model displays other phenotypes, including eye abnormalities, typically seen in patients with FA, and should thereby advance FA research on various fronts.

Since *SETD2* is an epigenetic factor that constrains elongation by RNAPs and regulates ribosomal biogenesis (47, 66–68), our *Setd2^+/–^ Fanca^–/–^* model appears to combine a source of increased R-loops and dsRNA with decreased resolution of these structures due to deficiency for *Fanca*. Here, we note that heterozygous KO of *SETD2* results in transcriptional dysfunction (69). Additionally, although we have combined the *Setd2^+/–^* mutation with a *Fanca*-KO mutation, our findings should be relevant to other complementation groups associated with components of the FA pathway, since they share a common DNA damage sensitivity when mutated (16).

On a separate note, *SETD2* is downregulated under inflammatory stress, which subsequently leads to the downregulation of multiple *FANC* genes (data not shown). In patients with FA, genetic modifiers or frequent infections during infancy may lead to *SETD2* downregulation, which could exacerbate the FA phenotype and contribute to the development of BMF. Thus, we posit that other modifiers, such as variant alleles or environmental stresses or stimuli, may contribute to the development of BMF in HSCs in patients with FA. In particular, the presence of *SETD2* in mice could act as a genetic modifier that elevates FA gene expression and may thereby explain why single FA KO mouse models do not display FA patient–like phenotypes. In line with this possibility, SETD2 expression is decreased in BM cells from patients with FA compared with healthy donors (Figure 1K). Further studies may help answer this question.

In addition to R-loop–associated genomic instability, multiple mechanisms have been proposed to contribute to the pathogenesis of BMF in FA (70, 71). Defective DNA interstrand cross-link repair is a hallmark of FA and leads to persistent DNA damage responses, chromosomal instability, and p53-mediated apoptosis in HSPCs (72). Chronic activation of inflammatory cytokines such as TNF-α and IFN-γ further exacerbates stem cell loss by inducing oxidative and replicative stress (73). Mitochondrial dysfunction and elevated ROS production have also been implicated in the progressive depletion of the HSPC pool (74). Moreover, studies have shown that replication stress and impaired recovery from stalled replication forks promote premature senescence and exhaustion of FA HSCs (75). Together, these findings suggest that FA-associated BMF results from a combination of DNA repair deficiency, inflammatory signaling, and metabolic stress. Our results place R-loop accumulation within this multifactorial context, indicating that targeting transcription-replication conflicts may complement existing strategies to mitigate hematopoietic failure in FA (76).

Our study of the cellular mechanisms involved in BMF in *Setd2^+/–^ Fanca^–/–^* mice has unveiled an apparent role for defects in the cell cycle and mitosis in FA-associated BMF. HSCs from these *Setd2^+/–^ Fanca^–/–^* mice, aged 4 weeks, displayed limited maintenance of a quiescent G0 phase, more mitotic errors, and reduced proliferation when cultured ex vivo. Importantly, in patients with FA, BMF often occurs during the early teenage years or at an even younger age (77). The equivalent in mice is an age of approximately 3 to 4 weeks (78). At these ages in mice and humans, HSCs are still in the cycling phase and have not yet entered the quiescent stage (79). Although not all *Setd2^+/–^ Fanca^–/–^* HSCs exhibit mitotic errors during cell division, the more rapid cell cycle in HSCs from younger mice increases the likelihood of mitotic errors. Importantly, the accelerated cell cycle and associated mitotic errors in HSCs from young *Setd2^+/–^ Fanca^–/–^* mice, or in pediatric or adolescent patients with FA, may lead to differentiation or cell death, and may thereby likely contribute to severe BMF.

We found that Juglone, an inhibitor of transcription by RNAP I, II, and III (51), maintains HSC survival by reducing R-loops, thereby potentially resolving ribosomal biogenesis defects. Here, we utilized Juglone to manipulate R-loop levels, via effects on RNAP-dependent transcriptional elongation. Juglone is a small compound found in all parts of walnut trees and other plants in the Juglans family. In human fibroblasts, juglone decreases p53 protein levels and induces H2A.X phosphorylation and cell death because of DNA damage and inhibition of transcription (80). Juglone has previously been used as an anticancer agent, with effective concentrations typically ranging from 1 to 10 μM. In contrast, our study utilized juglone at a significantly lower concentration (100 nM) to inhibit pan-RNAPs. Notably, this dose is approximately one-tenth of the concentration commonly employed for anticancer activity. In short-term in vitro culture, treatment with 100 nM juglone maintained the survival of control mouse HSCs and improved the survival of *Setd2^+/–^ Fanca^–/–^* HSCs (Figure 5, H and F). Furthermore, in long-term experiments, particularly after BM transplantation, juglone treatment rescued the chimerism of *Setd2^+/–^ Fanca^–/–^* BMCs and also reduced the chimerism of WT BMCs at 1 month after transplant (Figure 6J). To advance this discovery toward clinical application, further studies are required to assess the potential toxicity of juglone under various experimental conditions. The single cell–based cell division assay showed that Juglone treatment slows down the first cell division (Figure 6C). Furthermore, the CytoTell-based cell division history assay indicated that Juglone treatment reduces the number of cells with a medium division history and increases the number of cells with a low division history (Figure 6, D–G). These results suggest that Juglone treatment slows cell division, allowing HSCs sufficient time to prepare for the next cell division and to resolve R-loop and dsRNA accumulation. To clarify the effects of Juglone on mitotic errors in *Setd2^+/–^* *Fanca^–/–^* HSCs, a liquid culture assay was performed. Juglone-treated WT control HSCs did not show any notable difference compared with untreated controls; however, in *Setd2^+/–^* *Fanca^–/–^* HSCs, Juglone treatment significantly reduced mitotic errors (Figure 6H). Although Juglone has beneficial effects in this context, it is important to note that Juglone is known to be a cytotoxic compound due to its redox-cycling activity and generation of ROS (81). Excessive ROS production can lead to oxidative stress, DNA damage, and cell death, particularly at higher concentrations or with prolonged exposure. Therefore, the dose and exposure duration of Juglone must be carefully optimized to minimize cytotoxicity while preserving its protective effects on HSCs. Nevertheless, in this context, it should be noted that doses of Juglone that increased BMC and HSC numbers in vivo appear to have been tolerated in mice (Figure 6, L–N).

To explore potential therapies for BMF in FA, we hypothesized that reduction of R-loop accumulation could restore stemness in *Setd2^+/–^ Fanca^–/–^* HSCs. However, ectopic expression of RNASEH1, DICER, and SENATAXIN on *Setd2^+/–^ Fanca^–/–^* HSPCs did not rescue HSPC function, indicating that simply reducing R-loop and dsRNA levels is not by itself sufficient to rescue the function of *Setd2^+/–^ Fanca^–/–^* HSPCs (Supplemental Figure 3B). Indeed, treatments with L-leucine, low doses of the RNAPI inhibitor CX5461, and the pan-RNAP inhibitor Juglone all significantly reduced R-loop and dsRNA levels (Figure 5, A and B). However, only Juglone also improved cell survival in *Setd2^+/–^ Fanca^–/–^* HSCs (Figure 5, F–H), suggesting that a balanced inhibition of RNAPs may be necessary to restore HSC function. Thus, modulating the activity of RNAPs could be a promising therapeutic strategy for patients with FA. Importantly, low HSC numbers and HSC defects may be a barrier to autologous gene therapy for patients with FA, and although allogeneic HSC transplant is the frontline treatment for FA-related BMF, it also poses significant risks. Our study suggests that oral drug therapies targeting RNAPs, which thereby reduce R-loop/dsRNA accumulation, may ultimately relieve ribosomal biogenesis defects and make autologous transplants a safer alternative. The promising results from our in vitro treatments of HSCs with Juglone warrant further preclinical/clinical investigation and validation, including in vivo treatments in mice. A therapeutic strategy to prevent or cure BMF in FA, based on pan-inhibition of RNAPs with a drug such as Juglone, via rescue of HSC defects, may also potentially apply to other BMF conditions. Even more broadly, this approach may also help maintain HSCs ex vivo or facilitate HSC editing for many other applications.

## Methods

### Sex as a biological variable.

Sex was not considered as a biological variable in this study.

### Animals.

We bred *Fanca^+/–^* and *Setd2^+/–^* mice (47, 82) to generate *Setd2^+/–^ Fanca^–/–^* offspring on a C57BL/6 background where *Fanca*-KO mice develop reduced numbers of HSCs and craniofacial defects (83). All animal experiments were performed according to the guidelines of the IACUC.

### Cell preparation.

Whole BM cells were obtained by flushing the femur and tibia bones with DMEM (Sigma-Aldrich) containing 10% FBS (Biowest). The collected BM cells were then washed once with Dulbecco’s PBS (Sigma-Aldrich) containing 2% FBS. For HSC sorting, BM cells were first stained with a microbead-conjugated antibody for c-Kit (Miltenyi Biotec), and c-Kit^+^ cells were initially separated using an autoMACS Pro Separator (Miltenyi Biotec) before further staining with fluorescence-conjugated antibodies, lineages CD4 (RM4-5, BioLegend) CD8 (53-6.7, BioLegend), CD3 (17A2, BioLegend), CD11b (M1/70, BioLegend), Gr1 (RB6-8C5, BD Biosciences), B220 (RA3-6B2, BioLegend), and TER119 (TER-119, BD Pharmingen), along with CD117 (2B8, BioLegend), EPCR (eBio1560, eBioscience), CD48 (HM48-1, BioLegend), and CD150 (TC15-12F12.2, BioLegend).

### Cell culture.

Sorted E-SLAM HSCs were cultured with StemSpan (STEMCELL Technologies) treated with mouse thrombopoietin (100 ng/mL, PeproTech) and stem cell factor (100 ng/mL, PeproTech). For MMC treatment, E-SLAM HSCs were treated with different concentrations of MMC (0.01, 0.1, 1, 10 ng/mL) and cultured for 2 days. After 2 days of culture, HSC numbers were counted using Celigo (Revvity). For cell division assays, sorted E-SLAM HSCs were stained with CytoTell-green (AAT Bioquest) and cultured for multiple days, varying for each experiment.

### Single-cell paired-daughter assay.

Individual E-SLAM HSCs were initially cultured in StemSpan medium supplemented with murine stem cell factor and thrombopoietin (each at 100 ng /mL) for 48 hours. Subsequently, cells were transferred to IMDM containing 10% FBS along with murine SCF, TPO, G-CSF, EPO, and IL-3. After 14 days of culture, individual clones derived from LSK-SLAM cells were harvested and analyzed for differentiation into myeloid lineages, including neutrophils, erythrocytes, macrophages, and megakaryocytes.

### Transient transfections and lentiviral transductions.

Lentiviruses were generated by cotransfecting HEK293T cells with lentiviral vector plasmids (pLenti-IRES-eGFP, pLenti-RNASEH1-IRES-eGFP, or pLenti-DICER-IRES-eGFP) and packaging plasmids using calcium phosphate and collected virus at a 24-hour interval. HEK293T cells were maintained in DMEM with 10% FBS in a humidified incubator at 37°C and 5% CO_2_. c-Kit^+^ (CD117) cells collected from control or *Setd2^+/–^ Fanca^–/–^* mouse BM were transduced twice with collected lentiviruses using RetroNectin (Takara Bio).

### Antibodies for flow cytometry.

The following monoclonal antibodies were used as surface markers for cell sorting and flow cytometric analyses: anti-c-Kit (2B8), anti-CD150 (TC15-12F12.2), anti-CD48 (HM48-1), anti-EPCR (eBio1560; eBioscience), anti–Sca-1 (E13-161.7), anti-CD45.2 (104), anti-CD45.1 (A20), anti-B220/CD45R (RA3-6B2), anti–Mac-1 (M1/70), anti-Gr-1 (RB6-8C5), anti-CD4 (RM4-5), and anti-CD8 (53-6.72) antibodies. All antibodies were obtained from BioLegend unless otherwise noted. For R-loop and dsRNA detection, we utilized anti-DNA-RNA hybrid antibody (S9.6) (Kerafest) and dsRNA antibody (J2) (Cell Signaling Technology). Flow cytometric analysis and cell sorting were performed by using FACSCelesta (Becton Dickinson), FACSAria III (Becton Dickinson), and FACSMelody (Becton Dickinson), respectively. Obtained data were analyzed using FlowJo software (v. 10. 6. 1).

### Transplantation.

As described previously (57), C57BL/6 Ly5.1 recipient mice were lethally irradiated (total 9.5 Gy). Within 24 hours after irradiation, 1 × 10^6^ whole BM cells (test cells; Ly5.2) were transplanted with 1 × 10^6^ or 1 × 10^5^ whole BM cells (competitor cells; Ly5.1). At 1, 3, and 5 months after the transplantation, the chimerism in peripheral blood was analyzed using flow-cytometric analyses. For secondary transplantation, 5 months after the first transplantation, BM cells were harvested, and 1 × 10^6^ BM cells were transplanted to lethally irradiated (total 9.5 Gy) C57BL/6 Ly5.1 recipient mice. Five months after the secondary transplantation, peripheral blood chimerism was analyzed using flow-cytometric analysis.

### Confocal microscopy.

FA patient–derived cells were seeded into a chamber plate (Nunc). For HSC seeding, we used fibronectin coating. After cells were attached to the bottom of the chamber plate, they were fixed with 2% paraformaldehyde (Thermo Fisher Scientific) for 10 minutes and permeabilized with 0.1% Triton X-100 (Thermo Fisher Scientific) for 15 minutes, both at room temperature. After permeabilization, samples were blocked with 3% BSA in Tris-buffered saline with Tween 20 (TBST; Sigma-Aldrich) for 30 minutes. Then, samples were stained with primary and secondary antibodies in 3% BSA in TBST for 30 minutes at room temperature. Primary antibodies included S9.6 (ENH001, Kerafast), J2 (76651, Cell Signaling Technology), and Ubf1 (ab244287, Abcam). After secondary antibody staining, samples were incubated with DAPI (Invitrogen) for 10 minutes in TBST and mounted with ProLong Diamond Antifade Mountant (Invitrogen). After enclosing with cover glasses, samples were observed with an ECHO spinning disk confocal microscope.

### In vivo treatment with Juglone.

Juglone was diluted in a PEG-based vehicle. Mice were i.p. administered Juglone at a dose of 100 μg/kg body weight in a total volume of 20 μL per mouse.

### qPCR for ribosomal genes.

For mouse HSCs, total RNA was isolated using the RNeasy micro kit (QIAGEN) and converted to cDNA using SuperScript VILO (Invitrogen). The cDNA was amplified using an Applied Biosystems Step One Plus thermal cycler (Applied Biosystems). Specific probes for each gene were used and are listed in Table 1.

### Statistics.

Statistical analyses were performed using GraphPad Prism version 8.4.3. Data are presented as mean ± SEM unless otherwise indicated. Statistical significance was determined using a 1-tailed Student’s *t* test for comparisons between 2 groups or 1-way ANOVA with appropriate post hoc tests for multiple comparisons. A *P* value of less than 0.05 was considered statistically significant.

### Study approval.

All animal studies were conducted according to an approved IACUC protocol (20210066AR) and federal regulations at UT Health San Antonio.

### Data availability.

Values for all data points found in graphs are in the Supporting Data Values file.

## Author contributions

MH, TU, PRA, and GH designed the study and wrote the manuscript; MH performed most of the experiments; and XF and HZ performed patient-derived cell experiments. JB, JL, and TL helped harvest mice for some analyses.

## Funding support

MH by the EvansMDS Young Investigator Award (EPEF-N2023216-202320).
PA by US Department of Defense (W81XWH2210410).
GH by US Department of Defense (W81XWH2110148) and NIH R01 grants (CA248019 and CA266256).
Evans MDS DRG Grant (EPEF-DRG2020 to GH).
P30 Cancer Center Support Grant (CA054174 to GH).

## Supplementary Material

Supplemental data

Supporting data values

## Figures and Tables

**Table 1:**
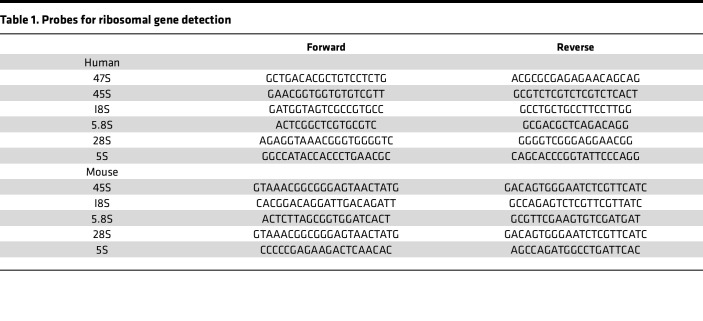
Probes for ribosomal gene detection

## Version 1 02/26/2026

In-Press Preview

## Version 2 04/08/2026

Electronic publication

## References

[1] Seita J, Weissman IL (2010). Hematopoietic stem cell: self-renewal versus differentiation. Wiley Interdiscip Rev Syst Biol Med.

[2] Nakamura-Ishizu A (2014). The analysis, roles and regulation of quiescence in hematopoietic stem cells. Development.

[3] Choi KD (2011). Hematopoietic differentiation and production of mature myeloid cells from human pluripotent stem cells. Nat Protoc.

[4] Kallen ME (2019). Acquired and germline predisposition to bone marrow failure: diagnostic features and clinical implications. Semin Hematol.

[5] Korthof ET (2013). Management of acquired aplastic anemia in children. Bone Marrow Transplant.

[6] Alter BP (2010). Malignancies and survival patterns in the National Cancer Institute inherited bone marrow failure syndromes cohort study. Br J Haematol.

[7] Shimamura A, Alter BP (2010). Pathophysiology and management of inherited bone marrow failure syndromes. Blood Rev.

[8] Dufour C (2017). How I manage patients with Fanconi anaemia. Br J Haematol.

[9] Schifferli A, Kuhne T (2015). Fanconi anemia: overview of the disease and the role of hematopoietic transplantation. J Pediatr Hematol Oncol.

[10] Mamrak NE (2017). Recent discoveries in the molecular pathogenesis of the inherited bone marrow failure syndrome Fanconi anemia. Blood Rev.

[11] Knies K (2017). Biallelic mutations in the ubiquitin ligase RFWD3 cause Fanconi anemia. J Clin Invest.

[12] Kolinjivadi AM (2020). Emerging functions of Fanconi anemia genes in replication fork protection pathways. Hum Mol Genet.

[13] Gille JJ (2012). Diagnosis of Fanconi anemia: mutation analysis by multiplex ligation-dependent probe amplification and PCR-based sanger sequencing. Anemia.

[14] Nalepa G, Clapp DW (2018). Fanconi anaemia and cancer: an intricate relationship. Nat Rev Cancer.

[15] Park JY (2016). Complementation of hypersensitivity to DNA interstrand crosslinking agents demonstrates that XRCC2 is a Fanconi anaemia gene. J Med Genet.

[16] Garcia-Higuera I (2001). Interaction of the Fanconi anemia proteins and BRCA1 in a common pathway. Mol Cell.

[17] Sims AE (2007). FANCI is a second monoubiquitinated member of the Fanconi anemia pathway. Nat Struct Mol Biol.

[18] Meetei AR (2003). A novel ubiquitin ligase is deficient in Fanconi anemia. Nat Genet.

[19] Rickman KA (2015). Deficiency of UBE2T, the E2 ubiquitin ligase necessary for FANCD2 and FANCI ubiquitination, causes FA-T subtype of Fanconi anemia. Cell Rep.

[20] Smogorzewska A (2007). Identification of the FANCI protein, a monoubiquitinated FANCD2 paralog required for DNA repair. Cell.

[21] Virts EL (2015). AluY-mediated germline deletion, duplication and somatic stem cell reversion in UBE2T defines a new subtype of Fanconi anemia. Hum Mol Genet.

[22] Alcon P (2020). FANCD2-FANCI is a clamp stabilized on DNA by monoubiquitination of FANCD2 during DNA repair. Nat Struct Mol Biol.

[23] Tan W (2020). Monoubiquitination by the human Fanconi anemia core complex clamps FANCI:FANCD2 on DNA in filamentous arrays. Elife.

[24] Ameziane N (2015). A novel Fanconi anaemia subtype associated with a dominant-negative mutation in RAD51. Nat Commun.

[25] Vaz F (2010). Mutation of the RAD51C gene in a Fanconi anemia-like disorder. Nat Genet.

[26] Wang AT (2015). A dominant mutation in human RAD51 reveals its function in DNA interstrand crosslink repair independent of homologous recombination. Mol Cell.

[27] Xia B (2007). Fanconi anemia is associated with a defect in the BRCA2 partner PALB2. Nat Genet.

[28] Reid S (2007). Biallelic mutations in PALB2 cause Fanconi anemia subtype FA-N and predispose to childhood cancer. Nat Genet.

[29] Sawyer SL (2015). Biallelic mutations in BRCA1 cause a new Fanconi anemia subtype. Cancer Discov.

[30] Howlett NG (2002). Biallelic inactivation of BRCA2 in Fanconi anemia. Science.

[31] Bluteau D (2016). Biallelic inactivation of REV7 is associated with Fanconi anemia. J Clin Invest.

[32] Bogliolo M (2013). Mutations in ERCC4, encoding the DNA-repair endonuclease XPF, cause Fanconi anemia. Am J Hum Genet.

[33] Kim Y (2011). Mutations of the SLX4 gene in Fanconi anemia. Nat Genet.

[34] Stoepker C (2011). SLX4, a coordinator of structure-specific endonucleases, is mutated in a new Fanconi anemia subtype. Nat Genet.

[35] Auerbach AD (2009). Fanconi anemia and its diagnosis. Mutat Res.

[36] Howlett NG (2005). The Fanconi anemia pathway is required for the DNA replication stress response and for the regulation of common fragile site stability. Hum Mol Genet.

[37] Andreassen PR (2004). ATR couples FANCD2 monoubiquitination to the DNA-damage response. Genes Dev.

[38] Ishiai M (2008). FANCI phosphorylation functions as a molecular switch to turn on the Fanconi anemia pathway. Nat Struct Mol Biol.

[39] Chen YH (2015). ATR-mediated phosphorylation of FANCI regulates dormant origin firing in response to replication stress. Mol Cell.

[40] Thompson EL (2017). FANCI and FANCD2 have common as well as independent functions during the cellular replication stress response. Nucleic Acids Res.

[41] Lossaint G (2013). FANCD2 binds MCM proteins and controls replisome function upon activation of s phase checkpoint signaling. Mol Cell.

[42] Liang Z (2019). Binding of FANCI-FANCD2 complex to RNA and R-loops stimulates robust FANCD2 monoubiquitination. Cell Rep.

[43] Garcia-Rubio ML (2015). The Fanconi anemia pathway protects genome integrity from R-loops. PLoS Genet.

[44] Schwab RA (2015). The Fanconi anemia pathway maintains genome stability by coordinating replication and transcription. Mol Cell.

[45] Yang S (2023). Helicases in R-loop formation and resolution. J Biol Chem.

[46] Allison DF, Wang GG (2019). R-loops: formation, function, and relevance to cell stress. Cell Stress.

[47] Zhou Y (2018). Setd2 regulates quiescence and differentiation of adult hematopoietic stem cells by restricting RNA polymerase II elongation. Haematologica.

[48] Skourti-Stathaki K (2014). R-loops induce repressive chromatin marks over mammalian gene terminators. Nature.

[50] Drygin D (2011). Targeting RNA polymerase I with an oral small molecule CX-5461 inhibits ribosomal RNA synthesis and solid tumor growth. Cancer Res.

[51] Chao SH (2001). Juglone, an inhibitor of the peptidyl-prolyl isomerase Pin1, also directly blocks transcription. Nucleic Acids Res.

[52] Vlachos A (2020). L-leucine improves anemia and growth in patients with transfusion-dependent Diamond-Blackfan anemia: results from a multicenter pilot phase I/II study from the Diamond-Blackfan Anemia Registry. Pediatr Blood Cancer.

[53] Paule MR, White RJ (2000). Survey and summary: transcription by RNA polymerases I and III. Nucleic Acids Res.

[54] Garaycoechea JI (2012). Genotoxic consequences of endogenous aldehydes on mouse haematopoietic stem cell function. Nature.

[55] Parmar K (2009). Mouse models of Fanconi anemia. Mutat Res.

[56] Meessen S (2022). A comparative assessment of replication stress markers in the context of telomerase. Cancers (Basel).

[57] Otsuki T WJ (1998). Assessment of mitomycin C sensitivity in Fanconi anemia complementation group C gene (Fac) knock-out mouse cells. Int J Hematol.

[58] Navarro S (2006). Hematopoietic dysfunction in a mouse model for Fanconi anemia group D1. Mol Ther.

[59] Sanij E (2008). UBF levels determine the number of active ribosomal RNA genes in mammals. J Cell Biol.

[60] Panov KI (2006). UBF activates RNA polymerase I transcription by stimulating promoter escape. EMBO J.

[61] Haeusler RA, Engelke DR (2006). Spatial organization of transcription by RNA polymerase III. Nucleic Acids Res.

[62] Vinciguerra P (2010). Cytokinesis failure occurs in Fanconi anemia pathway-deficient murine and human bone marrow hematopoietic cells. J Clin Invest.

[64] Gueiderikh A (2021). Fanconi anemia A protein participates in nucleolar homeostasis maintenance and ribosome biogenesis. Sci Adv.

[65] Pontel LB (2015). Endogenous formaldehyde is a hematopoietic stem cell genotoxin and metabolic carcinogen. Mol Cell.

[66] Walter DM (2023). Setd2 inactivation sensitizes lung adenocarcinoma to inhibitors of oxidative respiration and mTORC1 signaling. Commun Biol.

[67] Jiang Y (2022). Cross-regulome profiling of RNA polymerases highlights the regulatory role of polymerase III on mRNA transcription by maintaining local chromatin architecture. Genome Biol.

[68] Vlach L (2024). 51 Epigenetic regulation of ribosomal RNA transcription and processing by the tumor suppressor SETD2. Oncologist.

[69] Mitchell B (2023). Cellular and molecular functions of SETD2 in the central nervous system. J Cell Sci.

[70] Garaycoechea JI, Patel KJ (2014). Why does the bone marrow fail in Fanconi anemia?. Blood.

[71] Ceccaldi R (2016). The Fanconi anaemia pathway: new players and new functions. Nat Rev Mol Cell Biol.

[72] Kottemann MC, Smogorzewska A (2013). Fanconi anaemia and the repair of Watson and Crick DNA crosslinks. Nature.

[73] Pang Q (2001). FANCC interacts with Hsp70 to protect hematopoietic cells from IFN-gamma/TNF-alpha-mediated cytotoxicity. EMBO J.

[74] Pagano G (2012). Oxidative stress in Fanconi anaemia: from cells and molecules towards prospects in clinical management. Biol Chem.

[75] Ceccaldi R (2011). Spontaneous abrogation of the G_2_DNA damage checkpoint has clinical benefits but promotes leukemogenesis in Fanconi anemia patients. J Clin Invest.

[76] Sollier J, Cimprich KA (2015). Breaking bad: R-loops and genome integrity. Trends Cell Biol.

[77] Fink O (2023). Two decades of stem cell transplantation in patients with Fanconi anemia: analysis of factors affecting transplant outcomes. Clin Transplant.

[78] Delage C (2021). Traumatic brain injury: an age-dependent view of post-traumatic neuroinflammation and its treatment. Pharmaceutics.

[79] Hashimoto M (2021). Autophagy is dispensable for the maintenance of hematopoietic stem cells in neonates. Blood Adv.

[80] Paulsen MT, Ljungman M (2005). The natural toxin juglone causes degradation of p53 and induces rapid H2AX phosphorylation and cell death in human fibroblasts. Toxicol Appl Pharmacol.

[81] Ahmad T, Suzuki YJ (2019). Juglone in oxidative stress and cell signaling. Antioxidants (Basel).

[82] Rani R (2008). Differential p53 engagement in response to oxidative and oncogenic stresses in Fanconi anemia mice. Cancer Res.

[83] Wong JCY (2003). Targeted disruption of exons 1 to 6 of the Fanconi anemia group A gene leads to growth retardation, strain-specific microphthalmia, meiotic defects and primordial germ cell hypoplasia. Hum Mol Genet.

[84] Ruppitsch W (1997). The role of oxygen metabolism for the pathological phenotype of Fanconi anemia. Hum Genet.

