# Supplementary material for: Insights and modulation of RNA polymerase–dependent R-loop and dsRNA in Fanconi anemia hematopoietic stem cells
Source: JCI Insight. 2026 Feb 26;11(7):e192126. doi: 10.1172/jci.insight.192126 (PMC13134729; doi:10.1172/jci.insight.192126)
Supplement: Supplemental data [file jciinsight-11-192126-s264.pdf]

Supplemental Fig. 1

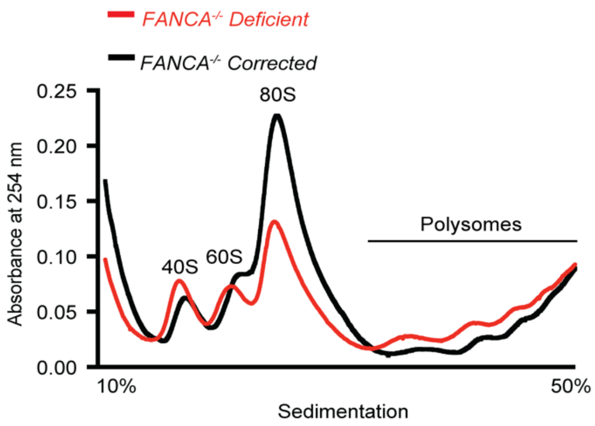

Supplemental Figure 1. *FANCA* deficiency reduces levels of the 60S subunit and mature 80S ribosomes. Cytosolic extracts were prepared from 20X10^6 cells from HSC72 FA-A pairs and sedimented by centrifugation on 10-50% sucrose gradients. Peaks corresponding to the 40S and 60S ribosomal subunits, 80S mono-ribosomes and ribosomes in polysome fractions were detected.

Supplemental Fig. 2

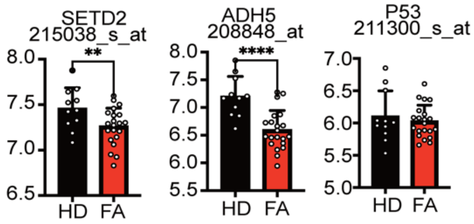

Supplemental Figure 2. Gene expression of FA patient low-density bone marrow cells.

Relative expression levels of SETD2, ADH5, and TP53 in healthy donors (HD, n = 11) and Fanconi anemia patients (FA, n = 21) were analyzed using normalized microarray data (GSE16334). Expression values are presented as log2-transformed intensities. Data are shown as mean ± SEM. Statistical significance was determined by one-tailed Student’s t test. *$p < 0.05$, **$p < 0.01$, ***$p < 0.001$, and ****$p < 0.0001$.

Supplemental Fig. 3

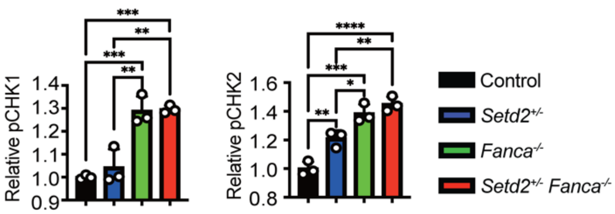

Supplemental Figure 3. Replication stress were accumulated in Setd2+/- Fanca-/- HSCs.

The levels of pCHK1 and pCHK2 in HSC from Control (black, n=3), *Setd2*$^{+/-}$ (Blue, n=3), *Fanca*$^{-/-}$ (Green, n=3) and *Setd2*$^{+/-}$ *Fanca*$^{-/-}$ (Red, n=3) mice. Data are shown as mean ± SEM. Statistical significance was determined by one-way ANOVA with Tukey’s multiple-comparison test. *p < 0.05, **p < 0.01, ***p < 0.001, and ****p < 0.0001.

Supplemental Fig. 4

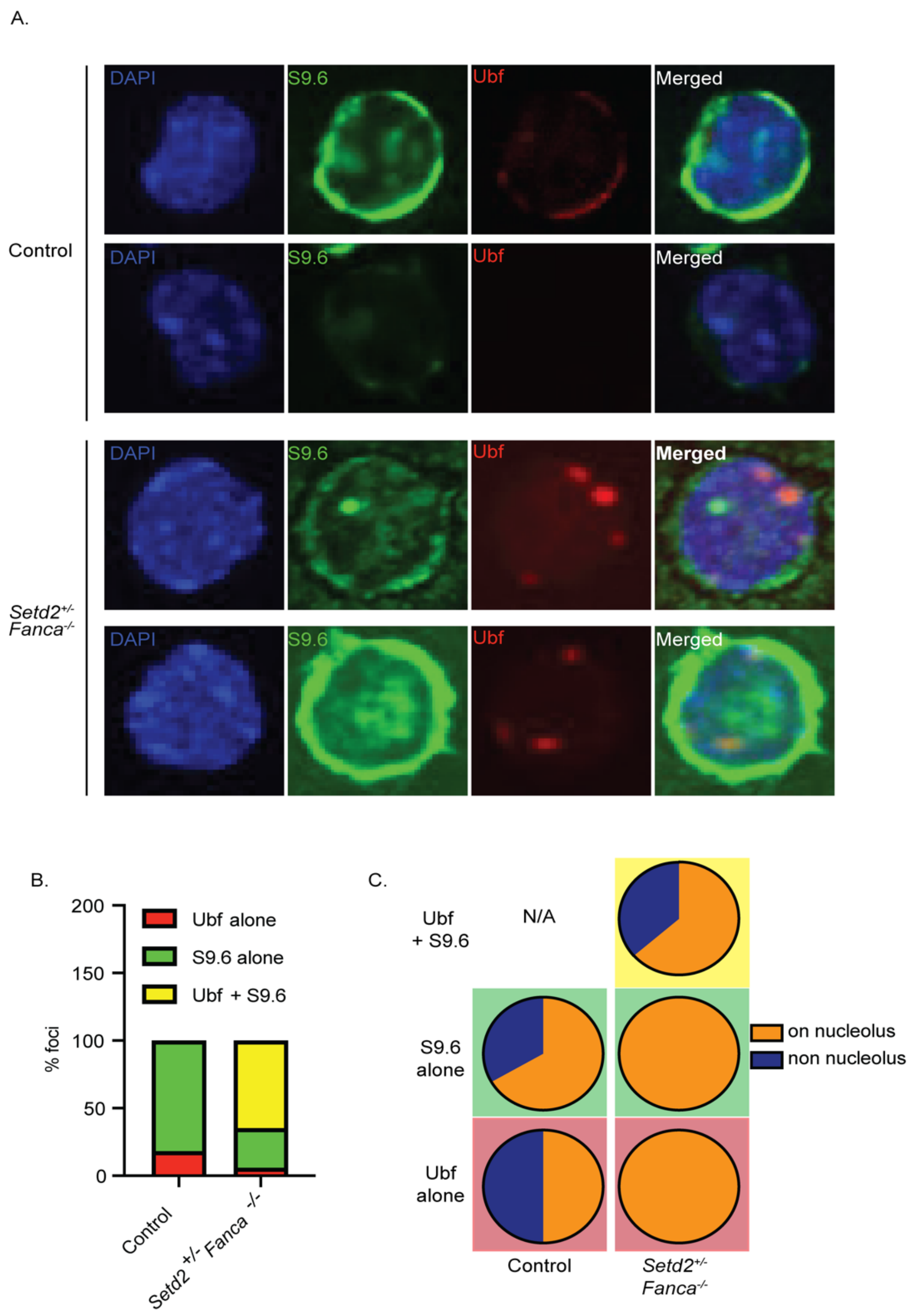

Supplemental Figure 4. R-loops accumulate at ribosome-related loci in nucleoli in *Setd2*$^{+/-}$ *Fanca*$^{-/-}$ HSCs.

(**A**) Additional image for visualization of colocalization of ribosomal protein and R-loops in HSCs. UBF (Red, Marker for RNA polymerase I), S9.6 (Green, R-loops), and DAPI (Blue). (**B**) Frequency of foci for UBF alone (Red), S9.6 alone (Green), and UBF and S9.6 colocalization (Yellow). (**C**) Frequency of focis (UBF alone (Red area), S9.6 alone (Green area), and UBF and S9.6 colocalization (Yellow area)) location on the nucleolus or not.

Supplemental Fig. 5

A.

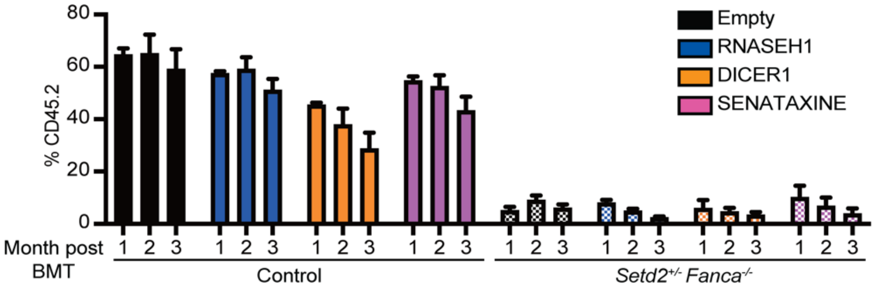

B.

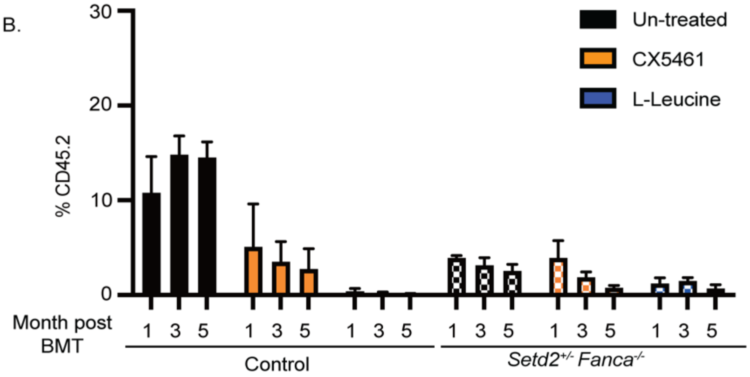

1037

Supplemental Figure 5. CX5461 and L-Leucine treatment do not rescue engraftment of *Setd2*$^{+/-}$ *Fanca*$^{-/-}$ HSPCs.

(A) Bone marrow transplantation of Control (no pattern), *Setd2*$^{+/-}$ *Fanca*$^{-/-}$ (pattern), with lentivirus-mediated transduction of Empty, *RNASEH1, DICER1, and SENATAXINE*. Peripheral bloods (PBs) were analyzed at 1, 2, and 4 months post-transplantation (SD; n = 5 in each condition). (B) Bone marrow transplantation of Control (no patern), *Setd2*$^{+/-}$ *Fanca*$^{-/-}$ (pattern), with or without treatment with CX5461 (yellow bar) and L-Leucine (Blue bar). PBs were analyzed at 1, 3, and 5 months post-transplantation (SD; n = 3 in each condition). Data are shown as mean ± SEM. Statistical significance was assessed using **two-way ANOVA** followed by **Tukey's multiple-comparisons test**. *p < 0.05, **p < 0.01, ***p < 0.001, and ****p < 0.0001.

Table 1

	Forward	Reverse
Human		
47S	GCTGACACGCTGTCCTCTG	ACGCGCGAGAGAACAGCAG
45S	GAACGGTGGTGTGTCGTT	GCGTCTCGTCTCGTCTCACT
18S	GATGGTAGTCGCCGTGCC	GCCTGCTGCCTTCCTTGG
5.8S	ACTCGGCTCGTGCGTC	GCGACGCTCAGACAGG
28S	AGAGGTAAACGGGTGGGGTC	GGGGTCGGGAGGAACGG
5S	GGCCATACCACCCTGAACGC	CAGCACCCGGTATTCCCAGG
Mouse		
45S	GTAAACGGCGGGAGTAACTATG	GACAGTGGGAATCTCGTTCATC
18S	CACGGACAGGATTGACAGATT	GCCAGAGTCTCGTTCGTTATC
5.8S	ACTCTTAGCGGTGGATCACT	GCGTTCGAAGTGTCGATGAT
28S	GTAAACGGCGGGAGTAACTATG	GACAGTGGGAATCTCGTTCATC
5S	CCCCCGAGAAGACTCAACAC	AGCCAGATGGCCTGATTCAC

Table 1. Probes for ribosomal gene detection.
